# A Review on Direct Electrochemistry of Catalase for Electrochemical Sensors

**DOI:** 10.3390/s90301821

**Published:** 2009-03-13

**Authors:** Periasamy Arun Prakash, Umasankar Yogeswaran, Shen-Ming Chen

**Affiliations:** Department of Chemical Engineering and Biotechnology, National Taipei University of Technology, No.1, Section 3, Chung-Hsiao East Road, Taipei 106, Taiwan (ROC); E-Mails: arunprakash.p@gmail.com (P.A.P.); uyogesh@gmail.com (U.Y.)

**Keywords:** Direct electrochemistry, catalase, electrocatalysis, hydrogen peroxide, electrochemical sensors, nanomaterials

## Abstract

Catalase (CAT) is a heme enzyme with a Fe^(III/II)^ prosthetic group at its redox centre. CAT is present in almost all aerobic living organisms, where it catalyzes the disproportionation of H_2_O_2_ into oxygen and water without forming free radicals. In order to study this catalytic mechanism in detail, the direct electrochemistry of CAT has been investigated at various modified electrode surfaces with and without nanomaterials. The results show that CAT immobilized on nanomaterial modified electrodes shows excellent catalytic activity, high sensitivity and the lowest detection limit for H_2_O_2_ determination. In the presence of nanomaterials, the direct electron transfer between the heme group of the enzyme and the electrode surface improved significantly. Moreover, the immobilized CAT is highly biocompatible and remains extremely stable within the nanomaterial matrices. This review discusses about the versatile approaches carried out in CAT immobilization for direct electrochemistry and electrochemical sensor development aimed as efficient H_2_O_2_ determination. The benefits of immobilizing CAT in nanomaterial matrices have also been highlighted.

## 1. Introduction

The study of the direct electron transfer pathways of redox proteins or enzymes is very significant in understanding the redox proteins, as well as in development of enzyme biosensors, biofuel cells and biomedical devices [1]. An in-depth study of the direct electrochemistry (DET) of heme enzymes provides satisfactory information regarding these bioelectrocatalysts (enzymes) and their major roles in electrocatalysis [2–6]. In the past decades, numerous works have been done to study the DET of various redox proteins and enzymes at modified electrodes. In 1977, a new gateway for protein direct electrochemistry was opened, when two groups, Yeh *et al*. and Eddowes *et al*. [7,8], independently reported the phenomenon of reversible electron transfer at a cytochrome c (cyt c) modified electrode. Since then, a large number of proteins and enzymes were successfully immobilized on a variety of biological and chemical matrices. Recently, Wolenberger *et al*. have reviewed direct protein electrochemistry using isolated enzymes and enzyme-protein couples [9]. The studies of redox proteins’ direct electrochemistry are thus important in understanding the mechanism of electron transfer between immobilized proteins and the electrodes.

On the other hand, an electrochemical sensor is a device in which a biochemical recognition process is coupled to an appropriate electrochemical transducer where one of the electrochemical transducer components is the electrode. To enhance the efficiency of the transducer, the electrode surfaces have been modified either using redox active compounds or bio-components like enzymes and proteins. Among enzymes, nearly 3,000 wild type enzymes are known and 1,060 of them are oxidoreductases. The oxidoreductase enzymes family are very important since they catalyze H_2_O_2_ reduction within the cells. During this reduction process, H_2_O_2_ is reduced to H_2_O and molecular O_2_ without forming free radicals. This reaction is mainly catalyzed by catalase (CAT) present in the red blood cells of mammals. CAT is a heme-containing redox protein with ferritoprotoporphyrin IX at its redox centre which belongs to the oxidoreductase family class [10]. It possesses a heme prosthetic group at its active site with metallic iron (Fe^III^). The catalytic ability of CAT to reduce H_2_O_2_ has been applied in the development of H_2_O_2_ sensors [11]. In order to investigate this catalytic ability of CAT in detail, the direct electrochemistry of CAT at the transducer surface has to be examined, but earlier studies showed that immobilization of CAT directly on the bare electrode surface lead to poor electron transfer [12]. This may be due to the fact that the Fe^(III/II)^ group gets deeply buried inside the huge structure of CAT. In order to achieve better electron transfer, CAT has been immobilized on various modified electrode surfaces. The electrode surfaces were modified either with surfactants, biopolymers, organic polymers, hydrogels, sol-gels or dendrimers, respectively [15–25]. Though noticeable electron transfer was observed with a few modified electrodes, the majority of them fail to promote the electron transfer process. This might be due to the diminished contact between CAT and such modified matrices. To overcome these problems, the bioelectric contact between CAT and the bare electrode was improved with the implementation of nanomaterials [29]. It is a well known fact that, with large surface area and highly porous network, nanomaterials are well suited for the entrapment of biological molecules [49–51]. Further, proteins or enzymes can be easily immobilized at these nanomaterials without any damage. In recent years, many researchers have attempted to immobilize CAT on various nanomaterial modified electrode surfaces. Their results ultimately illustrate that CAT immobilized on nanomaterial matrices possesses enhanced electron transfer and excellent catalytic activity. However, a survey of the literature shows that no one has presented a review on the direct electrochemistry of CAT and the influence of nanomaterials on the direct electron transfer of CAT. Consequently, in the present review we have discussed the direct electrochemistry of CAT on various modified electrode surfaces, both in the presence and absence of nanomaterials. This review mainly highlights the various electrode fabrication methods, their electrochemical characterizations, miscellaneous enzyme immobilization strategies used by researchers in the present and past decades, along with the benefits of using nanomaterials as enzyme immobilization matrices.

## 2. Nanomaterial Free Matrices for CAT Immobilization - Versatile Approaches

### 2.1. Polyelectrolyte Encapsulated CAT

**Figure 1.**
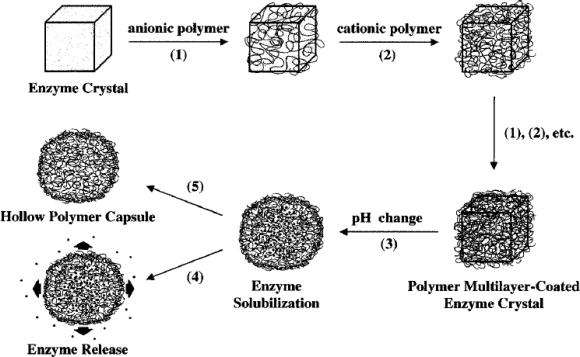
Schematic representation of the process involved in the encapsulation of CAT into polymer multilayers using biocrystals as templates. (Steps 1, 2) stepwise deposition of poly electrolyte layers; (Step 3) Exposure of the enzyme to solutions of pH > 6 or acidic solution (pH < 4), which results in the morphology change of the polymer capsule accompanied by the solubilisation of the enzyme; (Step 4) Exposure of the enzyme to solutions of pH > 11 leads to the release of the enzyme by rupturing of the polymer capsule; (Step 5) Exposure of the encapsulated enzyme to an oxidizing solution results in the decomposition of the enzyme and thus enzyme expelled from the interior of the capsule through the polymer walls, leaving behind the hollow polymer capsules (reproduced with permission from Caruso *et al. Langmuir* **2000**, *16*, 1485–1488).

Earlier attempts were made by researchers to immobilize CAT onto various nanomaterial free matrices. Caruso and co-workers carried out a simple yet versatile approach to encapsulate CAT crystals within polymer multilayer capsules via the stepwise regular assembly of oppositely charged polyelectrolytes, poly(styrenesulfonate) (PSS) (negatively charged) and poly(allylamine hydrochloride) (PAH) (positively charged), using biocrystals as templates [13]. The schematic representation of the process of enzyme encapsulation into the polymer films is shown in Figure 1. They examined the activity and stability of CAT present inside the polymer multilayer films through proteolysis. They observed that the polymer encapsulated CAT retained 100% of its activity, even after 100 min incubation with protease, whereas polymer uncoated CAT loses more than 90% of its initial activity within 100 min under the same conditions.

Yu *et al*. employed a similar approach to encapsulate CAT into polyelectrolytes, PAH and PSS respectively [14]. They investigated the direct electron transfer behavior and electrocatalytic response of polyelectrolyte encapsulated CAT to H_2_O_2_ reduction. The polyelectrolyte encapsulated CAT displays 4.5 times higher electrocatalytic response to H_2_O_2_ than non-encapsulated and solubilized CAT. However, the relative increase in catalytic response at the former was not proportional to the amount of enzyme deposited, since the entire CAT molecules were not found in their electrocatalytic active site. This might be due to the reason that the distance between the CAT molecules and the electrode surface gets significantly increased after polyelectrolyte encapsulation. Furthermore, they observed through their detailed studies that, with increases in precursor polyelectrolyte layer number, the current response to H_2_O_2_ decreased significantly, while the electrode response time increased. In contrast, implementation of fewer polyelectrolyte layers lead to sensitive and fast response in H_2_O_2_ determinations. This approach of encapsulating CAT micro crystals into polyelectrolyte layers for biosensing provides a versatile approach to prepare high enzyme content films with improved enzyme activities.

### 2.2. Surfactant Modified Matrices for CAT Immobilization

**Figure 2.**
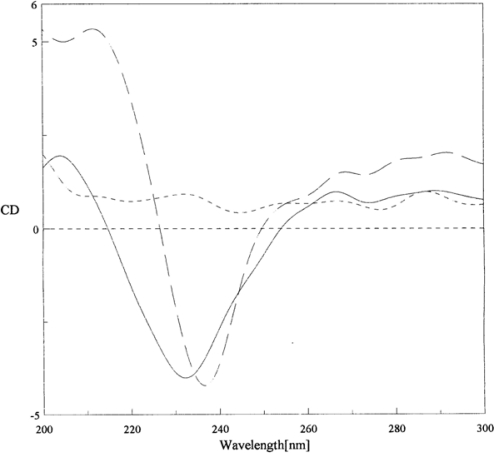
CD spectra of CAT (— —), DDAB (– – –), CAT-DDAB (—) films. CAT was prepared in pH 6.1, 10 mM phosphate buffer solution (PBS) with 50 mmol: l KCl. Temperature: 25°C (reproduced with permission from Chen *et al. Biosens. Bioelectron*. **2001**, *16*, 115–120)

In an alternative approach, Chen *et al*. attempted to immobilize CAT onto cationic surfactant, (didodecyldimethylammonium bromide, DDAB) liquid crystal films [15]. They revealed through their results that the electron transfer of CAT is greatly improved in the presence of DDAB. The electron transfer rate constant (*k*_s_) was observed to be 3.0 ± 0.4 s^−1^. However, their circular dichroism (CD) results illustrated that the conformation of CAT gets slightly perturbed by the hydrophobic nature of DDAB (Figure 2). The CD spectrum of CAT exhibits a negative peak at 237 nm, whereas for CAT-DDAB film it was observed at 232 nm. The negative peak at 232 nm was regarded as the shift from 237 nm of CAT film alone.

Gebica *et al*. also studied the interaction of the surfactant sodium bis(2-ethylhexyl) sulfosuccinate (AOT) with CAT and horseradish peroxidase (HRP) [16]. They studied comparatively the properties of CAT and HRP entrapped into AOT/*n*-heptane with those in aqueous solutions of AOT. Their detailed studies illustrate that the secondary structure of CAT changed significantly in the presence of aqueous micellar solutions of AOT and the activity of CAT was completely lost. Interestingly, the secondary structure of CAT remains unchanged in the AOT/*n*-heptane reverse micelles.

### 2.3. Immobilization of CAT at Various Polymers and Gel Matrices

**Figure 3.**
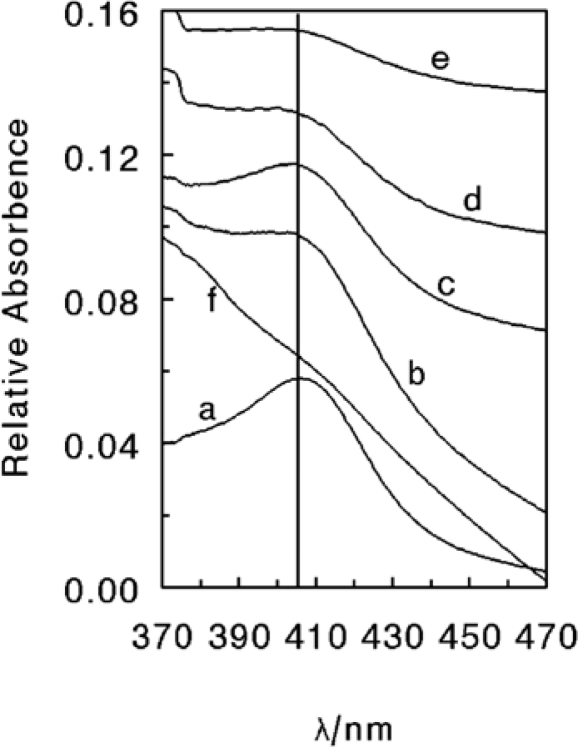
UV–vis absorption spectra of (a) dry CAT film, (b) dry CAT-PAM film, and CAT-PAM films in different pH buffer solutions: (c) pH 7.0; (d) pH 5.0; (e) pH 9.0 and (f) pH 3.0 (reproduced with permission from Lu *et al. Biophys. Chem*. **2003**, *104*, 623–632).

Other than surfactants, CAT has been immobilized in various matrices like biopolymers, organic polymers, hydrogels and sol-gels, respectively. Huang and co-workers attempted to immobilize heme proteins like myoglobin (Mb), hemoglobin (Hb), HRP and CAT into chitosan (CS) film modified pyrolytic graphite (PG) electrodes [17]. They obtained stable voltammogramms for MB, Hb and HRP films, whereas less stable voltammogramms were obtained in the case of CAT-CS films. Furthermore, compared to other protein-CS films, the electron transfer process also happens at a much slower rate in CAT-CS films and the stability was relatively very poor. Lu *et al*. have successfully immobilized CAT on polyacrylammide (PAM) hydrogel film-coated PG electrodes [18]. They investigated the direct voltammetric and electrocatalytic behavior of these CAT incorporated PAM films. They observed that the electron transfer of CAT was greatly facilitated in the microenvironment of PAM films. However, the electron transfer of these CAT-PAM films was much smaller and relatively less stable than CAT-CS films described above. Through UV–vis spectroscopy, they observed the position change of the Soret band for CAT-PAM films (Figure 3). They revealed through their results that CAT in dry PAM films retains a secondary structure that is the same as the native state of CAT in dry CAT films. Further, CAT in PAM films essentially retains its native state in buffers at medium pH. When the pH was more acidic or more basic, the Soret band showed a blue-shift with some distortion. As shown in Figure 3(f), substantial denaturation of CAT in PAM films takes place at pH 3.0. This illustrates the poor stability of CAT-PAM films at acidic pH values. Nevertheless, CAT-PAM shows good catalytic activity towards H_2_O_2_ reduction in the concentration range 0.4 – 0.8 mM (See Table 1).

Li *et al*. investigated the direct voltammetric behavior of heme proteins CAT and HRP immobilized on methyl cellulose (MC) modified edge-plane pyrolytic graphite electrodes [19]. Both CAT-MC and HRP-MC films displayed a good response to H_2_O_2_ reduction in the concentration range of 0.02 to 0.12 mM and 0.02 to 0.11 mM, respectively (See Table 1). However, the electron transfer rate observed on CAT-MC films was relatively lower than that of CAT-CS and CAT-PAM films. Li *et al*. also successfully immobilized CAT and Hb onto collagen modified PG electrodes [20]. Though they noticed direct electron transfer in these protein-collagen films, the redox peak potential of CAT-collagen films was found to be more negative than those of Hb-collagen films. This may be likely due to the reason that the larger polypeptide chains of CAT needs more active energy than Hb to electrochemically reduce at the PG electrodes. Moreover, Hb-collagen films were found to be more stable than CAT-collagen films. The reason for the greater stability of Hb-collagen films may be due to the electrostatic interactions between the oppositely charged groups present in collagen and Hb films. In addition, the hydrophobic and hydrogen-bond interactions between the collagen and Hb films further help to maintain the stability of these films. However, the reasons for the poor stability of CAT-collagen films were not reported. Further results show that, a more facile electron transfer process occurs at a much faster rate with Hb-collagen films than CAT-collagen films, which is obvious from the reported *k*_s_ values, (46 ± 6 s^−1^) and (31 ± 3 s^−1^), respectively.

**Figure 4.**
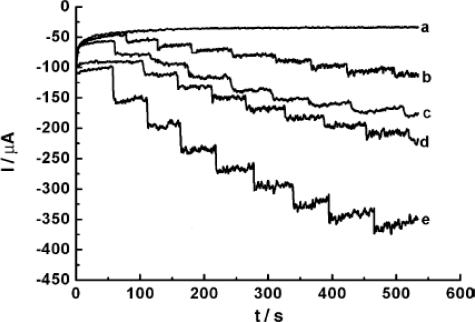
Amperometric i-t responses observed at different heme-proteins-SF/GCEs at − 0.2V in 10 ml of PBS pH 7.0 for 10μl injection of 22 mM H_2_O_2_ for every 80 s: (a) SF films; (b) Mb–SF films; (c) Hb–SF films; (d) CAT–SF films; and (e) HRP–SF films (reproduced with permission from Wu *et al. Anal. Chim. Acta* **2006**, *558*, 179–186).

Wu *et al*. investigated the direct electrochemistry of heme proteins Mb, Hb, HRP and CAT entrapped in silk fibroin (SF) films [21]. Among various protein-SF films, CAT-SF films relatively possess lower *k*_s_ value (0.34 s^−1^). This indicates that, much slower electron transfer process occurs at CAT-SF films. In contrast, the amperometric i-t measurement results reveal that, both HRP-SF and CAT-SF films were more active than Mb-SF and Hb-SF films towards the catalytic reduction of H_2_O_2_ (Figure 4).

From the calibration curves for H_2_O_2_ determination (not shown), the linear range of H_2_O_2_ was observed to be 0.004 to 0.085 mM for Mb–SF films, 0.003 to 0.091 mM for Hb–SF films, 0.002 to 0.354 mM for HRP–SF films and 0.003 to 0.158 mM for CAT–SF films, respectively.

In an alternative approach, Sun *et al.* immobilized heme proteins Hb, Mb and CAT into an organic polymer matrix, poly (N-isopropylacrylamide-co-3-methacryloxypropyltrimethoxysilane) (PNM) film coated glassy carbon electrode (GCE) [22]. All three protein-PNM films exhibit a facile electron transfer process, which is obvious from the obtained *k*_s_ values of 16, 17 and 20 s^−1^ for Hb-PNM, CAT-PNM and Mb-PNM films respectively. However, the stability of CAT-PNM films was much lower than Hb and Mb containing PNM films. CAT-PNM films show good catalytic activity towards H_2_O_2_ in the concentration range 0.002–0.035 mM (See Table 1).

**Figure 5.**
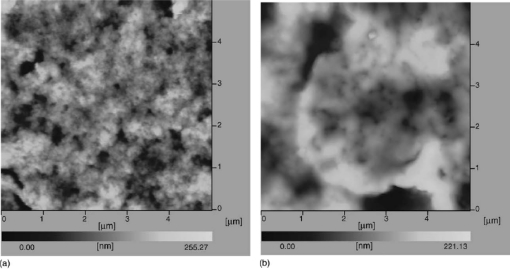
AFM images of: (a) Cysteine/Silica sol–gel and; (b) CAT/cysteine/Si sol–gel modified Au electrodes (reproduced with permission from Di *et al. Biosens. Bioelectron*. **2006**, *22*, 247–252).

The direct electrochemistry of CAT immobilized in silica sol-gel in the presence of cysteine on gold (Au) electrode was reported by Di *et al.* [23]. They confirmed the different surface morphology between silica sol-gel matrices with and without CAT through AFM studies. Figure 5 shows the AFM image of sol-gel matrix without CAT and with CAT. In their detailed study, they used this CAT/cysteine/Si sol–gel modified electrode for the determination of H_2_O_2_ in the range 0.001 to 0.03 mM with a detection limit of 0.4 μM, respectively (See Table 1). Further, they investigated the interaction of CAT and aluminium (Al^3+^) ions. They observed that, the electrocatalytic activity of CAT increased with the addition of Al^3+^ ions. Their detailed voltammetry and fluorescence spectroscopy results also confirms that, the addition of Al^3+^ ions leads to conformational changes in CAT molecule, which might be the reason for the improved catalytic activity of CAT.

Recently Wang *et al*. immobilized the heme proteins Hb, Mb and CAT into agarose hydrogel films [24]. They also investigated the effects of 1-butyl-3-methylimidazolium tetrafluoroborate ([bmim][BF_4_]) along with a small amount of water on the direct electrochemistry and bioelectrocatalysis of the entrapped heme proteins. Their CV results provide strong evidence that CAT entrapped in agarose films exhibits direct electron transfer and retains its electrochemical activity in [bmim][BF_4_] solution. A more facile electron transfer process occurs at CAT-agarose films (*k*_s_ = 49.1±9.3) than Hb-agarose (*k*_s_ = 36.1±7.3) and Mb-agarose (*k*_s_ = 29.1±7.0) films respectively. Further, CAT-agarose films show good catalytic response towards H_2_O_2_ in the concentration range 0.001 – 0.818 mM (See Table 1). The Michaelis-Menten constant (*K*_m_) for CAT-agarose films (317±77 μM) is two folds smaller than Hb and Mb, which indicates that the CAT entrapped in agarose film exhibits a higher affinity for H_2_O_2_.

### 2.4. Dendrimer Matrices for CAT Immobilization

Shen *et al.* successfully used a layer-by-layer assembly technique to immobilize Hb, Mb and CAT with polyamidoamine (PAMAM) dendrimers on different solid surfaces [25]. As well known, at pH 7 protonated PAMAM possesses positive surface charges, whereas the proteins have net negative surface charges. Thus, layer-by-layer {PAMAM-protein}_n_ films were assembled with alternate adsorption of oppositely charged PAMAM and proteins from their aqueous solutions mainly by electrostatic interaction. The assembly process was monitored by QCM, UV-vis spectroscopy and CV. The growth of the protein multilayer films was regular and linear, whereas the electroactivity of the films extended only to a few bilayers. CVs of {PAMAM-protein}_n_ films showed a pair of well-defined and nearly reversible peaks which are characteristic of the heme Fe^(III)/(II)^ redox couples of the corresponding proteins. Although {PAMAM-Hb}_n_ and {PAMAM-Mb}_n_ films showed very similar properties, {PAMAM-CAT}_n_ films displayed dissimilar properties. The substrates with biological or environmental significance, such as oxygen, H_2_O_2_, trichloroacetic acid, and nitrite, were catalytically reduced at {PAMAM-protein}_n_ film electrodes. This shows the potential applicability of the {PAMAM-protein}_n_ films as new types of biosensors or bioreactors. Both the electrochemical and electrocatalytic activity of {PAMAM-protein}_n_ films can be tailored precisely by controlling the number of bilayers or the film thickness. Among various protein-PAMAM films, CAT incorporated PAMAM films showed poor stability and the catalytic response towards H_2_O_2_ was limited only to two layers. The previous experimental results showed that greater the distance between the enzyme and the electrode surface lesser will be the stability. The CAT with greater molecular dimension (diameter 10.5 nm) also experience weaker interactions with PAMAM, which could be partly responsible for the less stability of {PAMAM-CAT}_n_ films.

As discussed in Section 2, CAT immobilized on various nanomaterial-free matrices possesses unique characteristics like facile electron transfer, good catalytic activity, fast response and appreciable stability. More immense evolution in the characteristics of CAT based sensors has been achieved in the recent years with the implementation of various nanomaterial matrices. The utilization of nanomaterials as CAT immobilization matrices narrowed the difficult tasks that existed over the past decades in the achievement of direct electrochemistry. Besides these, the long term stability of such nanomaterial-incorporated CAT films has promoted their wide applications in various fields. The significant advancements in the implementation of nanomaterial matrices for CAT immobilization towards the development of efficient H_2_O_2_ sensors are explained in detail in the subsequent section.

## 3. Nanomaterial Matrices used for CAT Immobilization

The direct electrochemistry of CAT at various nanomaterial modified matrices is discussed in the following sections.

### 3.1. CAT Immobilized in Single-wall Carbon Nanotubes (SWCNTs) Matrices

SWCNTs with high tensile strength and unique ability to grow on various electrode surfaces have advanced their applications in electrochemical sensor development. Recently, Salimi *et al*. reported the lowest ever detection limit of 10 pM determination of H_2_O_2_ with an excellent sensitivity (47.6 nA pM^−1^) at an NAD^+^ strongly adsorbed SWCNTs modified electrode [26]. This shows the excellent application of SWCNTs matrices towards the direct determination of H_2_O_2_. Moreover, the exceptional properties of SWCNTs have made them extremely suitable for enzyme immobilization and thus they are exclusively used for enzyme based biosensor development [27,28]. Interestingly, Wang and co-workers reported the direct electrochemistry of CAT at SWCNTs-Au electrode surface [29]. Their detailed electrode fabrication procedure involves the following steps: about 1 mg of purified SWCNTs was dissolved in 10 mL of *N, N*-dimethylformamide and the whole mixture was ultrasonicated until a uniform dispersion was obtained. About 15 μL of this SWCNTs suspension was casted on a clean Au electrode surface and dried under an infrared lamp. This SWCNTs-Au electrode was then immersed into 50 μM CAT in pH 5.9 PBS and kept for 20 min. For better performance, the CAT concentration was optimized as 8 μM to 80 μM respectively.

**Figure 6.**
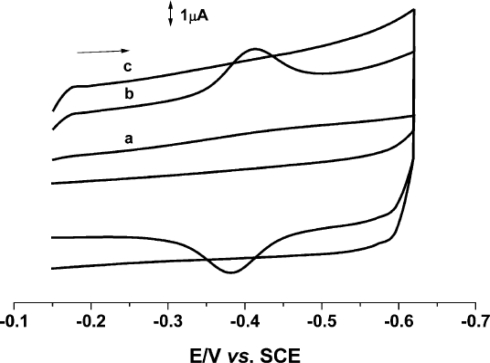
(a) CVs of CAT (adsorbed from a 50 μM CAT solution) at a bare Au electrode (b) SWCNTs-Au modified electrode with CAT and (c) SWCNTs-Au modified electrode without CAT. The electrolyte solution used was 0.05 M PBS pH 5.9 and the scan rate employed was 0.1 V s^−1^ (reproduced with permission from Wang *et al. Electroanalysis* **2004**, *16*, 627–632).

Their CV results illustrate that CAT adsorbed on bare Au electrode surface exhibits no significant redox peaks (Figure 6a). However, the SWCNTs-Au modified electrode with CAT exhibits a pair of well defined quasi reversible peaks at −0.414 V vs. SCE with a peak potential separation (ΔEp) value of 32 V (Figure 6b). Though in the absence of CAT, no noticeable peaks were observed for the SWCNTs modified Au electrode, considerable back ground current was found (Figure 6c). This provides evidence that the surface area of the Au electrode has been significantly increased in the presence of SWCNTs and this indeed enhanced the electron transfer process of CAT. Moreover, from their pH studies it was obvious that the catalytic peak current of CAT negatively shifted with pH 3.1 to 8.9 with a slope of 36 V. This reveals that the Fe ^(III/II)^ redox couple of CAT involves a one proton and two electron transfer process.

Moreover, their catalytic activity studies show that the CAT immobilized on SWCNT matrices exhibits a good response towards H_2_O_2_ in the concentration range 0.7 mM to 1.1 mM, respectively. All the above studies show that CAT has been successfully immobilized on SWCNTs, and the direct electron transfer and catalytic activity are greatly enhanced in the presence of SWCNTs. The analytical data have been given in Table 1.

### 3.2. CAT Immobilized in a SWCNTs-CS Matrix

CS is a multifunctional biopolymer which possesses exceptional properties like excellent film-forming ability, good adhesion, biocompatibility, high mechanical strength and has thus been used to immobilize biomolecules in recent years [30–33]. In order to increase the contact area between enzyme and SWCNTs, for better sensor sensitivity and stability, CS was widely employed to wrap SWCNTs. [34]. Jiang *et al*. observed that the incorporation of CS into SWCNTs lead to significant enhancements in the mechanical strength, tensile strength and conductivity of SWCNTs [35]. They have successfully immobilized CAT onto this SWCNTs-CS composite matrix and thereby observed the direct electrochemistry of CAT. In their detailed electrode fabrication process, SWCNTs were dispersed in 0.5% by weight CS solution and sonicated until a 0.5 mg mL^−1^ black suspension was obtained. About 5 μL of this SWCNT suspension was casted onto a cleaned GCE surface with a microsyringe, and dried at room temperature. The SWCNTs-GCE was immersed overnight in 10 mg mL^−1^ CAT solution at 4°C.

**Figure 7.**
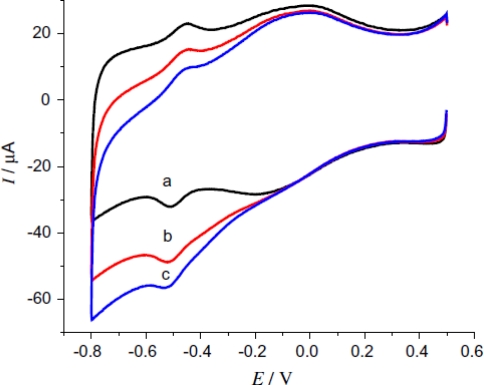
CVs of SWNTs-CS-CAT-GCE in PBS pH 7 at a scan rate of 0.1 V s^−1^ in the presence of (a) 0, (b) 1, and (c) 4 mM H_2_O_2_ (reproduced with permission from Jiang *et al. J. Electroanal. Chem*. **2008**, *623*, 181–186).

CV studies show that SWCNTs-CS-CAT-GCE exhibits a pair of well defined redox peaks at ca. −0.45 V and −0.5 V. The different scan rate studies explain that a surface confined process occurs at the modified electrode surface. From the average surface coverage concentration (Γ) value of CAT (16.4 nmol cm^−2^) they confirmed that a monolayer of CAT has been formed over the SWCNTs modified electrode. The pH studies show that a one electron/one proton coupled reaction occurs at SWNTs-CS-CAT-GCE. These results clearly illustrate that direct CAT electrochemistry has occurred in the SWCNTs-CS matrix. They further observed through their CV studies that SWNTs-CS-CAT-GCE exhibits a steady increase in cathodic reduction peak current at −0.50 V for every substantial addition of H_2_O_2_ to the analyte solution (Figure 7). The linear concentration range was observed to be 5–50 mM H_2_O_2_. The sensitivity was 6.32 μA mM^−1^cm^−2^ and detection limit was 2.5 μM, respectively (see Table 1). The following catalytic mechanism explains in detail about the electrocatalysis process that occurs at the electrode.:

(1) $${\text{Heme Fe}}^{\left(\text{III}\right)}+H_{2}O_{2}\to \text{Compound I}+H_{2}O$$

(2) $$\text{Compound I}+{2H}^{+}+{2e}^{-}\to {\text{Heme Fe}}^{\left(\text{III}\right)}$$

The electron transfer process becomes much more facile at SWCNTs platform, which was obvious from the very high *k*_s_ value of 118 s^−1^ respectively. The *k*_s_ value observed at SWCNTs-CS-CAT-GCE was relatively higher than the nanomaterial free matrix modified electrodes [15,20–22,24] reported in Section 2.

### 3.3. CAT Immobilized in Nafion-nano Gold-MWCNT Matrices

Zhou *et al.* reported the direct electrochemistry of CAT on multiwalled carbon nanotubes (MWCNTs) and gold nanoparticles (GNPs) modified pyrolitic graphite electrode (PGE) [36]. MWCNTs and GNPs provided an extremely stable microenvironment for the entrapped CAT molecule. Colloidal Au was prepared with slight modification according to the procedure of Frens *et al*. [37], and MWCNTs was pretreated to introduce carboxylic acid groups on the surface of CNTs [38]. The pretreated MWCNTs were dispersed in 1 mg mL^−1^ of 1% Nafion (NF) solution. The NF-CAT-GNP-MWCNTs-PGE was prepared by the following procedure: Initially a 6 mL mixture of MWCNTs and 1% NF solution was dropped on the surface of a cleaned PGE and dried at room temperature. Another 6 mL of a mixture of CAT (10 mg of CAT in 1 mL of 0.1M pH 6.98 PBS) and GNP (v = v ¼ 1:1) solution was dropped above the MWCNTs and NF surface and dried at ambient temperature without light. For stability, NF-CAT-GNP-MWCNTs-PGE was covered with a thin layer of 4 mL of 1% NF solution. Similarly NF-CAT-PGE and NF-CAT-MWCNTs-PGE were prepared as mentioned above.

**Figure 8.**
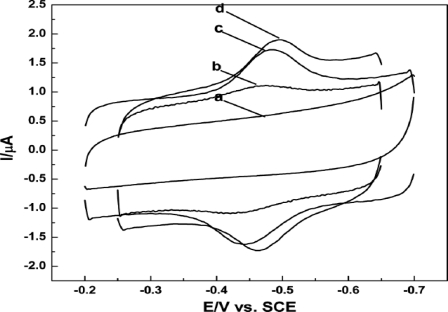
CVs of (a) NF-GNP-PGE, (b) NF-CAT-PGE, (c) NF-CAT-GNP-PGE (d) NF-CAT-GNP-MWCNTs-PGE electrode in 0.1 M pH 6.98 PBS at scan rate of 100 mV s^−1^ (reproduced with permission from Zhou *et al. Anal. Lett*. **2008**, *41*, 1832–1849).

The effect of GNPs on microstructures of CAT was examined through UV-vis spectroscopy results. An intense band at 280 nm with similar site/shape was observed for both CAT solution and CAT-GNP mixture. This in turn confirms that CAT in the mixture retains its native structure without the loss of biological activity. CV studies explain that NF-CAT-GNP-MWCNTs-PGE displays a pair of well-defined and nearly reversible redox peak in 6.98 PBS (Figure 8c). The anodic peak potential (Epa), cathodic peak potential (Epc) and formal potential (E^0^) at NF-CAT-GNP-MWCNTs-PGE was observed to be −0.461, −0.495 V and −0.478 V, respectively. From the slope of cathodic peak currents vs. scan rate, Γ value of CAT was found to be about 2.4 nmol cm^−2^. Electrochemical impedance spectroscopy (EIS) results show that both GNP-PGE and MWCNTs-PGE exhibits two obvious semicircles with smaller diameter, which provides strong evidence that GNP and MWCNTs promoted the electron transfer between CAT and PGE. As reported by Zhang *et al*, the interaction between the amino groups of enzyme and GNPs greatly facilitates the electron transfer process [39]. The transfer coefficient (α) and *k*_s_ of CAT was observed to be 0.49 and 1.387 s^−1^ (±0.1), respectively. This observed *k*_s_ value was relatively lower than that reported at nanomaterial-free matrices [20–22,24]. However, it was higher than CAT-SF films [21]. NF-CAT-GNP-MWCNTs-PGE also exhibits a high stability on storage. When stored at −20° C for 20 days the reduction peak current decreased only to 8%.

Further, the NF-CAT-GNP-MWCNTs-PGE exhibits an enhanced catalytic activity towards H_2_O_2_ and the catalytic current increased with increase in H_2_O_2_ concentration. The linear response of the modified electrode was 1–5 mM (See Table 1). The *K*_m_ value was observed to be 1.71 mM (±0.05), which in turn implies that the modified PGE exhibits higher affinity towards H_2_O_2_.

### 3.4. CAT Immobilized on MWCNTs Incorporated Glassy Carbon Electrodes

Salimi *et al*. successfully immobilized CAT on a MWCNTs modified GCE [40]. The detailed electrode modification procedure of MWCNTs-CAT modified GCE is illustrated as follows: prior to each experiments, 15 cycle scans were performed on a the clean GCE in the potential range of −2.0 to +2.0 V vs. SCE in a solution of 1 M H_2_SO_4_ [41]. About 25 μL of 0.4 mg mL^−1^ of acetone-MWCNTs solution was casted on the GCE and dried in air. In order to obtain a well defined stable CV, the resulting MWCNTs-GCE was dipped in 0.5 mM of CAT in 0.1 M of PBS solution and subjected to consecutive potential cyclic scans in the potential range of −1.0 to +1.5 V (scan rate 100 mV s^−1^). Finally, the modified GCE was removed from CAT solution and washed with double distilled water and stored in buffer at pH 7 at 4 °C before use.

The direct electrochemistry of CAT immobilized on MWCNTs modified GCE was investigated through CV. A pair of well defined and nearly reversible redox peaks were observed at −0.05 V in PBS 6.5 for MWCNTs-CAT-GCE. The formal potential E^0′^ of MWCNTs-CAT-GCE was ultimately more positive than the other electrodes [42–44] and approximately between 350–400 mV. The surface coverage (Γ) value of CAT (24 nmol cm^−2^) confirms the existence of a CAT monolayer on the MWCNTs electrode surface. A slope value of 61 mV pH^−1^ obtained through pH studies shows that the redox reaction of CAT was single electron coupled single proton transportation process [45]. MWCNTs-CAT-GCE also possesses a good stability on storage. When stored at 4 °C for 10 days, it shows a similar catalytic response and the reduction peak currents decreased only to 3%. The α and *k*_s_ values are 0.42 and 80 s^−1^, respectively. Thus, the *k*_s_ value observed at MWCNTs-CAT-GCE was comparatively higher than that reported at nanomaterial free matrices [15,20–22,24] and lower than that reported at nanomaterial modified matrices [36]. With the addition of H_2_O_2_, MWCNTs-CAT-GCE exhibits an obvious catalytic reduction peak at −0.25V. The catalytic peak current increased linearly with increase in H_2_O_2_ concentration in the range of 0.01 to 1 mM. The correlation coefficient was 0.9889 and the detection limit was 50 μM at a signal-to-noise ratio of 3. Amperometric i-t measurements show that MWCNTs-CAT GCE shows steady state response to H_2_O_2_ at −0.3 V for a linear concentration range 0.01 to 0.1 mM. The sensitivity was 3.3 nA μM^−1^ cm^−2^. The correlation coefficient was 0.9998 and the detection limit was 1 M, respectively (See Table 1). Moreover, MWCNTs-CAT-GCE exhibits greater affinity towards H_2_O_2_ which was obvious from the observed *K*_m_ value, 1.7 mM.

Zhou and co-workers also succeeded in immobilizing CAT onto MWCNTs modified GCEs. They investigated the direct electrochemistry of CAT in 0.1M tris(hydroxymethyl)aminomethane (Tris)–HCl buffer solution [46]. Interestingly, they reported the effect of La^3+^ ions on the electrocatalytic activity of CAT. Their results demonstrate that the electrochemical reaction of the immobilized CAT was promoted by low concentration of La^3+^ (0.001 mM) and inhibited by the high concentration (0.01 mM) of La^3+^. This might be due to the reason that low concentration of La^3+^ increases the non planarity and the exposure extent of CAT heme group and vise versa. Moreover, the CAT modified electrode retains its catalytic activity and exhibits a good response to H_2_O_2_ concentration in the concentration range 1 to 4.8 mM respectively. The *k*_s_ value was observed to be 1.0 7 s^−1^. This was much lower than that reported at some nanomaterial modified matrices [36,40] and nanomaterial free matrices [20,22,24].

**Figure 9.**
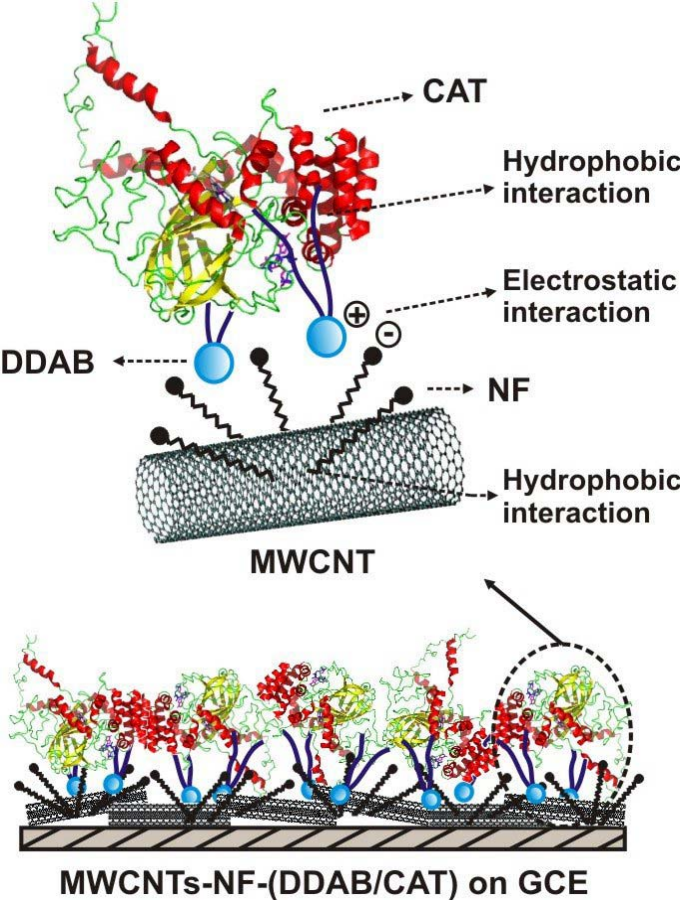
The possible interaction between MWCNTs, NF, DDAB and CAT in MWCNTs-NF-(DDAB/CAT) film modified electrodes. (Reproduced with permission from Prakash *et al. Talanta*, doi:10.1016/j.talanta.2009.02.033, article in press).

Recently we reported the direct electrochemistry of CAT on a NF dispersed MWCNTs modified GCE both in the presence and absence of DDAB [47]. We revealed through our studies that the presence of DDAB in the MWCNTs-NF-CAT film produced a 48% enhancement in the surface coverage concentration of CAT (Fe^III/II^) and 66% enhancement in *k*_s_ value, whereas the CAT stability increased by 57 %. We also reported a maximum *k*_s_ value of 11 s^−1^ at MWCNTs-NF-(DDAB/CAT) film modified GCE. Thus the *k*_s_ value observed at MWCNTs-NF-(DDAB/CAT) film modified GCE was comparatively higher than that observed at nanomaterial free matrices [15,21] and nanomaterial modified matrices [36,46]. Moreover, the sensitivity values of MWCNTs-NF-(DDAB/CAT) film for H_2_O_2_ reduction was calculated using CV and i-t curve measurements and it was about 35.62 and 101.74 μA mM^−1^ cm^2^ respectively (See Table 1). The possible interactions between MWCNTs-NF, DDAB and CAT at MWCNTs-NF-(DDAB/CAT) film on GCE have been given in Figure 9.

### 3.5. Covalent Immobilization of CAT on Conductive Composite Nanofiber Meshes

Wang et al. co-immobilized CAT on a neat poly(acrylonitrile-co-acrylic acid) (PANCAA) nanofiber mesh using 1-ethyl-3-(3-dimethylaminopropyl) carbodiimide hydrochloride (EDC) and *N*-hydroxy-succinimide (NHS) [48]. They also immobilized CAT on PANCAA and MWCNTs incorporated composite matrices. Their detailed enzyme immobilization procedure was described here. About 2 mg of the pretreated PANCAA/MWCNTs nanofiber mesh was soaked into 50 mM EDS/NHS (1:1) PBS and shaken gently for 6 h at room temp and thus activated. This activated nanofiber mesh was later mixed with CAT (0.10 mg mL^−1^ PBS) at room temp for a suitable time. Their results show that the activity of CAT on composite nanofiber matrix was 42 % higher than that of neat PANCAA nanofiber matrices. Furthermore, the electrical conductivity and viscosity of PANCAA/MWCNTs were superior to that of PANCAA alone. This may be due to the interaction between -COOH groups of etched MWCNTs and DMF and -COOH groups of PANCAA through hydrogen bond. The *K*_m_ value for free CAT, CAT/PANCAA and CAT/PANCAA/MWCNTs composite nanofiber mesh were observed to be about 34.07, 77.9 and 58.14 mM, respectively. The reasons for the higher *K*_m_ value of CAT at PANCAA and PANCAA/MWCNTs matrix might be due to conformational changes of CAT and the formation of a substrate-CAT complex. Furthermore, the increased limitation in diffusion also lessens the accessibility of the substrate to the active sites of immobilized CAT and thus promotes the *K*_m_ value.

**Figure 10.**
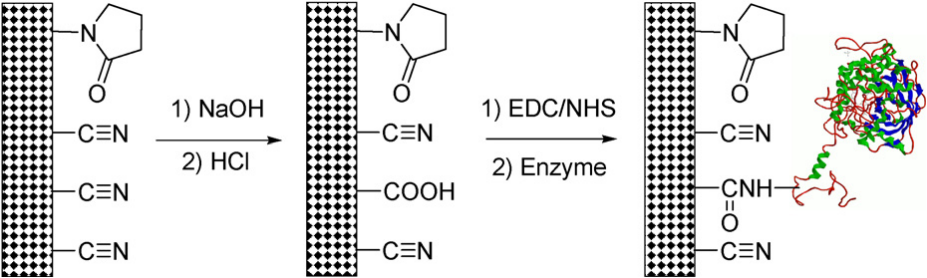
The two steps involved in the CAT immobilization are (Step 1) Activation of nanofibrous membrane through alkali treatment and (Step 2) Enzyme immobilization using EDC/NHS (reproduced with permission from Wan *et al. Enzyme Microb. Technol.* **2008**, *42*, 332–339).

Similarly, Wan *et al*. used poly(acrylonitrile-co-*N*-vinyl-2-pyrrolidone) (PANCNVP) and polyacrylonitrile (PAN) electrospun nanofibrous membranes with and without MWCNTs for the efficient immobilization of CAT [49]. The enzyme immobilization procedure involves two steps as explained in Figure 10. The first step involves the alkali treatment of the electrospun nanofibrous membranes, which results in the generation of -COOH groups. The second step was the activation step and it involves the treatment of nanofibrous membranes with EDC/NHS. The FESEM and TEM results confirm that presence of MWCNTs in the nanofibers increased the surface area, number of carboxylic groups and surface roughness. Further studies show that the presence of MWCNTs in the nanofibrous membranes increased the amount of immobilized CAT. The maximum amount of CAT was immobilized on PAN/MWCNTs (29.81±3.76 mg g^−1^ fibres) and PANCNVP/MWCNTs (25.77±4.10 mg g ^−1^ fibres) composite nanofibrous membranes, respectively. MWCNTs presence increased the activity retention and electrical conductivity of the nanofibres. Thus, CAT immobilized at PANCNVP/MWCNTs membrane possesses maximum activity retention (48.7 %), whereas PAN/MWCNTs possess a little lower activity retention (45.3%). Compared to free CAT, nanofibrous membrane immobilized CAT possesses lower, maximum reaction rates (Vmax) and higher *K*_m_ values. Their detailed stability studies also elucidate that nanofibrous membrane immobilized CAT possess improved stability. Further the immobilized CAT remains more biocompatible especially in the presence of MWCNTs. Thus among various nanofibrous membranes, MWCNTs containing nanofibrous membranes are most suited for CAT immobilization.

**Figure 11.**
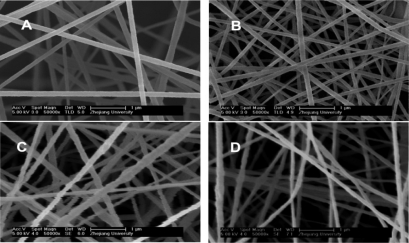
FESEM images of the electrospun nanofibers: (a) PAN (154±30 nm); (b) PANAACoPP (180±30 nm); (c) PAN/CNT (180±34 nm); (d) PANAACoPP/CNT (165±37 nm) (reproduced with permission from Wan *et al. J. Phys. Chem. C* **2007**, *111*, 14091–14097).

In an alternative approach, Wan and co-workers covalently immobilized CAT onto electrospun nanofibres (dia = 180 nm) of acrylonitrile-based copolymers containing porphyrin pendants, either in the presence or absence of MWCNTs [50]. The terpolymer, PANAACoPP was prepared from acrylonitrile, acrylic acid and metalloporphyrin with Co^2+^. Then nanofibers were fabricated from this terpolymer using an electrospining technique. The field emission scanning electron microscopy (FESEM) images obtained for various nanofibers both in the presence and absence of MWCNTs are shown in Figure 11. They clearly display that MWCNTs present in PAN/MWCNT and PANAACoPP/MWCNT nanofibres increased the roughness of the nanofibres. Moreover, due to the lower surface tension of the polymer solution, MWCNTs were mostly covered with thin polymer film and thus not much exposed to air.

Their detailed studies show that the nanofibers with MWCNTs contain greater amounts of CAT than without MWCNTs. For instance, in the absence of MWCNTs, one gram of PAN fibers contains only 24.45±2.33 mg CAT, whereas they contain 29.81±3.76 mg CAT in the presence of MWCNTs. Similarly, one gram of PANAACoPP nanofibres has only 18.93±4.03 mg CAT, whereas PANAACoPP/MWCNTs contain more CAT (22.81±4.22 mg). The specific activity and retention activity also increased in the presence of MWCNTs, however, the *K*m value decreased and V_max_ increased in the presence of MWCNTs. This clearly shows that blending of nanofibres with MWCNTs has significantly increased the characteristics of CAT in the nanofiber matrices. Their further studies indicate that both the introduction of porphyrin pendants and MWCNTs obviously improve the activity and stabilities of the immobilized CAT. The reason for the improvement in activity and stability might be due to the enhanced electron transfer between the immobilized CAT molecules and the ability of both porphyrin pendants and MWCNTs to retain the active conformation of the CAT.

### 3.6. CAT Immobilized on Nickel Oxide Nanoparticles Modified Glassy Carbon Electrodes

**Figure 12.**
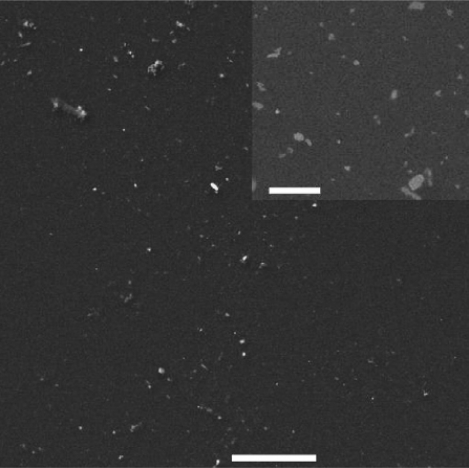
SEM image of the electrodeposited NiO on GCE, scale bare 20 μm. Inset is the SEM image with higher magnification for the same sample, scale bar is 2 μm (reproduced with permission from Salimi *et al. Biophys. Chem*. **2007**, *125*, 540–548).

Salimi and co-workers reported the direct electrochemistry of CAT on nickel oxide (NiO) nanoparticles modified GCE [51]. The NiO nanoparticles modified GC and indium tin oxide (ITO) electrodes were prepared as follows: initially Ni (−1.0 V, 5 min deposition time) was electrodeposited on a GCE using 1 mM nickel nitrate pH 4 acetate buffer solution. Then they repetitively cycled (30 scans) from 1.5 to −1.0 V at scan rate 100 mV s^−1^ in fresh PBS solution for electrodissolution and passivation of NiO layer. They determined the effective area of the modified electrode as 0.033 cm^2^ from CVs of 10 mM K_4_ [Fe(CN)_6_] in buffer solution at pH 7. They employed the same procedure for electrodeposition of NiO nanoparticles on ITO. Figure 12 shows the scanning electron microscopy (SEM) image of NiO film electrochemically deposited at GCE. This shows that the particle size of NiO varied from 100 nm to 700 nm. The larger particles are shown at the bottom right. Similarly, the immobilization of CAT on GCE and ITO modified with nano-scale islands of NiO was carried out using the following procedures. CV was used for immobilization of CAT on NiO nanoparticles with a one step method.

After the deposition of metallic Ni on GCE, the electrode was immersed in fresh PBS pH 7 solution containing 5 mg mL^−1^ CAT and the potential was repetitively cycled (30 scans) from +1 to −0.5 V at scan rate 100 mV s^−1^ for electrodissolution and passivation of NiO layer and immobilization of CAT. They used the same procedure to immobilize CAT and NiO on an ITO electrode. The CAT films on NiO exhibits a pair of well defined, stable and nearly reversible CV peaks at about −0.05 V vs. SCE at pH 7. E^0^′ of CAT in NiO film linearly varied in the range 1–12 with slope of 58.426 mV pH^−1^, indicating that the electron transfer is accompanied by single proton transportation. Moreover, α and *k*_s_ values for NiO-CAT-GCE were observed to be 0.45 and 3.7 s^−1^ (±0.1), respectively. The reason for more facile electron transfer process at NiO-CAT-GCE was due to the larger surface area and more porous structure of NiO nanoparticles which helps for the better entrapment of CAT. Further, NiO acts as a molecular wire and enhances the direct electron transfer of CAT. Their detailed studies illustrate that the chemical stability of NiO film and the interaction between CAT and NiO NPs have enhanced the overall stability of modified electrode. NiO-CAT GCE on 10 days storage (4 °C) showed no shift in CV potentials and the reduction peak currents decreased only to 2%.

The CAT embedded in NiO nanoparticles shows excellent electrocatalytic activity towards H_2_O_2_ reduction with a linear range from 0. 1 to 5.0 mM. The detection limit was 10 μM with a signal to noise ratio of 3. NiO-CAT modified GCE also exhibits a fast amperometric response within 2 s to H_2_O_2_ in the linear concentration range 0.001 – 1.0 mM. The sensitivity was observed to be 15.9 nA μM^−1^ and the correlation coefficient was 0.9988 respectively. The detection limit was found to be 0.60 μM. Thus, NiO-CAT modified GCE possess an excellent stability, long life and good reproducibility towards H_2_O_2_ determination. The apparent *K*_m_ value was 0.96 mM (±0.05), which shows the excellent catalytic activity of CAT incorporated NiO films towards H_2_O_2_. Moreover, exceptional electrochemical reversibility of redox couple, high stability, technical simplicity, lack of need for mediators and short preparation times are advantages of this NiO-CAT modified electrode.

## 4. Benefits of Nanomaterial Matrices above Nanomaterial Free Matrices for Efficient CAT Immobilization towards the Development of CAT Based Electrochemical Sensors

Miscellaneous CAT immobilization techniques, detailed electrode fabrication procedures along with various electrochemical and morphological characterizations both in the absence and presence of nanomaterials have been presented in Sections 2 and 3. The electrochemical parameters obtained through CV and i-t curve measurements for various nanomaterial modified electrodes were compared with that of nanomaterial free electrodes and given in Table 1. It was obvious from this Table that excellent electrocatalytic activity of CAT with very high sentivity and low detection limit was more pronounced in nanomaterial matrices rather than nanomaterial free matrices. In addition, the results discussed in Sections 2 and 3 clearly show that nanomaterial modified matrices are superior to nanomaterial free matrices in several aspects. Being extremely miniaturized in size and having large surface areas, nanomaterials are an excellent choice for CAT immobilization. CAT immobilized on those nanomaterial matrices remains extremely compatible and undergoes facile electron transfer processes. With excellent mechanical strength and exceptional conductivity, SWCNTs are more appropriate for CAT immobilization. As discussed in Sections 3.1 and 3.2, the presence of SWCNTs greatly facilitates the electron transfer process. Moreover, the *k*_s_ value for CAT at SWCNT-CS matrix was observed to be 118 s^−1^. It was comparatively higher than any of the nanomaterial modified and nanomaterial-free electrodes discussed before. Further, the detection limit, linear range and sentivity calculated at CAT-SWCNTs-CS modified electrode are given in Table 1. This in turn confirms the good catalytic ability of this SWCNTs modified electrode. Other than SWCNTs, the stability and activity of CAT greatly improved at MWCNTs matrices. This might be due to the biocompatible microenvironment of MWCNTs, which facilitates the immobilized CAT to retain its activity. Moreover, in the presence of MWCNTs the surface area and roughness largely increased. As described in Section 3.5, the activity retention and electrical conductivity extensively improved in the presence of MWCNTs. Moreover, the large surface area and porous nature of MWCNTs surfaces assists in the entrapment of more CAT. The electron transfer also becomes facile at MWCNTs matrices. It was obvious that the maximum peak current and lesser peak separation of CAT redox couples was observed at MWCNTs matrices. Furthermore, the electrocatalytic activity of CAT also considerably enhanced at MWCNTs matrices (see Table 1). MWCNTs-CAT modified GCE exhibits good catalytic activity towards H_2_O_2_. The lowest detection limit was observed to be 1 μM H_2_O_2_ and the sensitivity was 3.3 μA mM^−1^ cm^−2^, respectively. Other than, MWCNTs, NiO nanoparticles modified GCE also possess promising catalytic activity. The NiO-CAT-GCE exhibits a very high sensitivity of 15.9 μA mM^−1^cm^−2^ with 0. 6 μM H_2_O_2_ as lowest detection limit. The linear range of H_2_O_2_ concentration was about 0.001–1.0 mM respectively. Thus among various nanomaterials, CNTs and CNT incorporated matrices are excellent choices for better CAT immobilization and for the achievement of facile direct electrochemistry.

## 5. Conclusions

The versatile approaches practiced in the immobilization of CAT onto various matrices for the achievement of direct electron transfer have been reviewed. For this purpose CAT has been immobilized on various modified electrode surfaces both in the presence and absence of nanomaterials. All the CAT modified films were electrochemically characterized and their catalytic efficiency towards H_2_O_2_ was also investigated. The results illustrate that CAT incorporated nanomaterial matrices show outstanding characteristics like facile electron transfer, high sensitivity, low detection limit and excellent stability. Thus they are superior to CAT incorporated nanomaterial free matrices in several aspects. Although some nanomaterial free matrices promoted the direct electrochemistry of CAT, the majority of them slow down the electron transfer process. This might be due to the lesser contact noticed between CAT and those nanomaterial free matrices. On the other hand, most of the nanomaterials acted as nanowires and helped for the achievement of better contact between CAT and the electrode surface. This ultimately facilitated the electron transfer process. In the midst of various nanomaterial matrices, more fascinating direct electron transfer process was observed at CNTs modified matrices. Moreover, with large surface area and numerous pores on their surfaces, CNTs matrices have excellent ability to trap large amount of CAT molecules. CNTs incorporated CAT also exhibits excellent catalytic activity towards H_2_O_2_ and ultimately helps for the CAT based electrochemical sensors development. The future aspects of CAT based sensors depend on the development of novel nanomaterials with some advantageous properties. This perhaps helps for the better immobilization and significant enhancement in the direct electrochemistry of CAT and other CAT based redox enzymes and protein direct electrochemistry.

## Figures and Tables

**Table 1.** The electrochemical parameters of various CAT-modified electrodes calculated through CV and amperometric i-t studies.

**Modified Electrode**	**Techniques**	**E_pa_^a^ (or) E_pc_^b^ (V)**	**Linear range (mM)**	**Sensitivity (μA mM^−1^ cm^−2^)**	**Detection limit (μM)**	**Ref**
CAT-PAM	CV	−0.5^b^	0.4 – 0.8	-	-	[18]
CAT-MC	CV	−0.4^b^	0.02–0.12	-	-	[19]
CAT-SF	i-t	−0.2^b^	0.003–0.158	-	-	[21]
CAT-PNM	i-t	−0.25^b^	0.002–0.035	-	-	[22]
CAT/cysteine/Si sol–gel	i-t	0.1^b^	0.001–0.03	-	0.4	[23]
CAT-agarose	CV	−0.24^b^	0.001 – 0.818	-	-	[24]
CAT-SWCNTs	CV	−0.4^a^	0.7–1.1	-	4.0	[29]
CAT-SWCNTs-CS	CV	−0.52^b^	5–50	6.32	2.5	[35]
NF-MWCNTs-CAT–GNP	CV	−0.29^a^	1–5	-	-	[36]
CAT-MWCNTs	i-t	−0.3^b^	0.01–0.1	3.3	1.0	[40]
CAT-MWCNTs	CV	−0.56^b^	1.0 – 4.8	-	-	[46]
MWCNTs-NF-(DDAB/CAT)	CV	−0.38^b^	0.5 – 1.2	35.62	150	[47]
CAT-NiO	i-t	−0.3^b^	0.001–1.0	15.9	0.60	[51]

a Anodic peak potential (Epa)

b cathodic peak potential (Epc)
